# PPARs in Rhythmic Metabolic Regulation and Implications in Health and Disease

**DOI:** 10.1155/2010/243643

**Published:** 2010-09-07

**Authors:** Purin Charoensuksai, Wei Xu

**Affiliations:** McArdle Laboratory for Cancer Research, University of Wisconsin, Madison, WI 53706, USA

## Abstract

The circadian rhythm, controlled by a complex network of cellular transcription factors, orchestrates behavior and physiology in the vast majority of animals. The circadian system is comprised of a master clock located in central nervous system with 24-hour rotation and periphery clocks to ensure optimal timing of physiology in peripheral tissues. Circadian expression of peroxisome proliferator-activated receptors (PPARs), members of the nuclear receptor superfamily and key mediators of energy homeostasis and metabolism, is regulated by clock genes. PPARs serve as sensors of nutrient and energy/metabolism status to temporally entrain peripheral clock. Metabolism and circadian clocks are tightly intertwined: clock genes drive metabolism, and various metabolic parameters affect clock genes, producing a reciprocal feedback relationship. Due to PPARs' robust relationship with energy status and metabolism, the aberration of PPARs in the biological clock system leads to abnormal expression of genes in metabolic pathways, thus, contributing to etiology of metabolic syndrome. Studying PPARs' functions under the context of the mammalian circadian system could advance our understanding of how energy and metabolic status are maintained in the body, which may ultimately lead to rhythmic medical treatment against metabolic syndrome.

## 1. Introduction

### 1.1. The Mammalian Circadian Clock

The behavior and physiology of the vast majority living organisms oscillate in a 24-hour cycle in response to the light-dark cycle. It is estimated that 3%–20% of mammalian genes are under circadian regulation in a tissue-dependent manner [1]. Gene expression microarray analysis of mouse prefrontal cortex revealed that approximately 10% of transcripts demonstrate a diurnal expression rhythm [2]. Among the rhythmic genes identified, many have functions in biosynthetic and metabolic processes [1]. In addition to rhythmic transcriptional regulation of gene expression, various biological processes including hormone secretion, blood pressure, sleep-awake pattern, blood glucose and lipid level, body temperature, and metabolism exhibit circadian oscillation [3]. This endogenous response has clear implications in health and disease. For instance, the occurrence of certain cardiovascular diseases such as stroke, unstable angina, and myocardial infarction is markedly more frequent in specific parts of the day [4–6].

In mammals, the internal biological clocks are composed of central and peripheral components whose function is to coordinate biological processes to maintain synchrony with the environmental cycles of light and nutrients [7–9]. The central clock or master clock is located in the suprachiasmatic nuclei (SCN) of the anterior hypothalamus [10]. The role of the central clock is to coordinate peripheral oscillators situated in various peripheral tissues such as the liver, the kidney, the heart, and muscles in such a way that normal circadian rhythm is maintained at the organismal level [10, 11].

**Figure 1:**
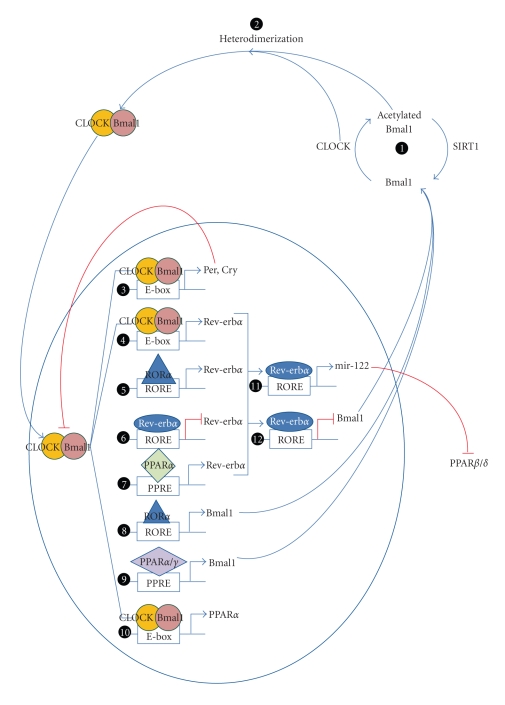
Regulatory networks of the core clock components and PPARs. (1) Bmal1 is acetylated by CLOCK which possesses acetyltransferase activity. This process can be reversed by SIRT1 deacetylase activity. Acetylated Bmal1 and CLOCK proteins heterodimerize (2) translocate into nucleus and activate transcription of Per, Cry (3), and Rev-erb*α* (4). In turn, Per and Cry form a repression complex which, upon translocation from the cytoplasm into the nucleus, inhibits transcription driven by CLOCK/Bmal1, including its own, constituting the main feedback loop. Bmal1 expression is controlled by Rev-erb*α* and ROR*α*, the two primary players in the secondary loop, in an opposing manner. Upon binding to a common RORE, Rev-erb*α* suppresses transcription (12) while ROR*α* exerts transcriptional activation of Bmal1 (8). The expression of Rev-erb*α* is driven by CLOCK/Bmal1 (4) and ROR*α* (5) and suppressed by itself (6). PPARs and the core clock proteins reciprocally regulate each other. PPARs regulate the transcription of some clock genes, for example, PPAR*α* activates Rev-erb*α* (7) and Bmal1 (9) while PPAR*γ* only activates Bmal1 transcription (9). On the other hand, clock genes regulate expression levels of PPARs. CLOCK/Bmal1 drives PPAR*α* expression (10) and Rev-erb*α* activates transcription of mir-122 (11), a microRNA which downregulates expression of PPAR*β*/*δ* posttranscriptionally.

**Figure 2:**
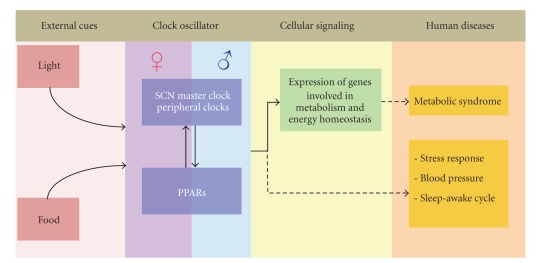
Overview of the functional link between the biological clocks and PPARs in health and disease. The external cues such as food and light can program SCN and peripheral clocks, leading to corresponding orchestrated expression of genes involved in metabolism and energy homeostasis. The gene products of clock components and PPARs reciprocally regulate each other, while both exhibit gender difference. Aberration in the biological clock-PPARs network is causative of metabolic syndrome and may also be responsible for human disorders including stress response, blood pressure, and sleep-awake cycle.

Light is a major daily external photic resetting signal of the circadian system in mammals. Through the retino-hypothalamic tract, light resets the master clock in the SCN triggering neural and humoral signaling that subsequently synchronizes the peripheral clock [8]. Although the pathways to synchronize an organism at the systemic level remain poorly understood, a model to explain this event at the cellular level has been established. Transcription factors, activators, and repressors are now emerging as clock components. They form feedback regulatory loops resulting in rhythmic expression of each component and lead to cascades of gene expression with 24-hour periodicity. The key players are two transcription factors: brain and muscle Arnt-like protein 1(Bmal1) and CLOCK/NPAS2 [7], which form heterodimers to constitute the central loop (Figure 1, (2)). CLOCK has intrinsic acetyl acetyltransferase transferase activity [8] and acetylates its heterodimer partner Bmal1 [8]. The acetylated Bmal1/CLOCK heterodimer then binds to the E-box (5′-CACGTG-3′) enhancer elements of Period genes (Per1, Per2, and Per3) and Cryptochrome gene (Cry1 and Cry2) promoters (Figure 1, (3)) [8, 12, 13] to activate transcription of Per and Cry [14]. The products of these genes interact to form repressor complexes which translocate into the nucleus and inhibit Bmal1-CLOCK/NPAS2 activity, thus resulting in repression of their own transcription [7]. To sustain the negative feedback loop, multiple components are subjected to posttranslational regulations. For example, Per and Cry proteins are regulated by ubiquitin-proteasome pathways [15], and CLOCK acetyltransferase activity can be reversed by SIRT1 deacetylase (Figure 1, (1)) [8].

In addition to central transcription factors regulating the central loop, another feedback loop line tunes the activity of the core clock components (Figure 1) [16]. Bmal1 expression is controlled by orphan nuclear receptors: retinoid-related orphan receptor alpha (ROR*α*) and Rev-erb*α* (NR1D1) [7, 8]. ROR*α* and Rev-erb*α* are closely related and recognize similar response elements (RORE) [7]. As a result, they compete for common RORE in the Bmal1 promoter and trigger opposing responses [7]. ROR*α* interacts with peroxisome proliferator-activated receptor-*γ* coactivator-1*α* (PGC-1*α*) resulting in histone acetylation and subsequent Bmal1 transcription activation (Figure 1, (8)) whereas Rev-erb*α* interacts with corepressors leading to histone deacetylation and Bmal1 transcription inhibition (Figure 1, (12)) [14]. This ROR*α*/Rev-erb*α* interplay is believed to be responsible for Bmal1 rhythmic expression. Reciprocally, Bmal1 and CLOCK regulate Rev-erb*α* by binding to the E-box in Rev-erb*α* promoter (Figure 1, (4)) [7]. Moreover, the promoter of Rev-erb*α* also contains RORE rendering it to be negatively controlled by itself and positively controlled by ROR*α* (Figure 1, (5) and (6)) [7]. 

A recent study by Schmutz et al. has identified a novel role of Per2 in the regulation of circadian rhythm; that is acting as cofactors for two nuclear receptors. Per2 was shown to interact with Rev-erb*α* at Bmal1 promoter to repress Bmal1 expression in liver in a Rev-erb*α*-dependent manner [17]. In the liver of *Rev-erb* *α* ^−/−^/*Per2* mutant mice, Bmal1 rhythmic mRNA expression was completely abolished [17].In a different phase, Per2 can be brought to the PPRE region of Bmal1 promoter by PPAR*α* and functions as a coactivator to enhance Bmal1 expression. Taken together, Per2 appears to be a modulator of Bmal1 expression by two circadian mechanisms: rhythmic repression mediated by Rev-Erb*α* and rhythmic activation mediated by PPAR*α*.

Attempts have been made to identify novel regulators of the biological clocks. A recent study employing genome-wide RNAi screening has identified ~1000 genes of which knockdown resulted in amplitude reduction, and hundreds of genes of which knockdown altered oscillation period length or increased amplitude [18]. Pathway analysis of newly identified genes has shown that many of these genes participate in insulin signaling, hedgehog signaling, cell cycle regulation, and folate metabolism [18], exemplifying the involvement of biological clocks in the regulation of cellular metabolism, growth, and development. Several reviews have discussed the molecular mechanisms of biological clocks in great details [3, 8, 13], thus are referred to for further reading. 

### 1.2. PPARs Overview

Peroxisome proliferator-activated receptors (PPARs) are a subfamily of the nuclear receptor superfamily of transcriptional factors [19]. The PPAR subfamily constitutes three members: PPAR*α*, PPAR*β*/*δ* and PPAR*γ* [19–21]. Despite a high degree of homology observed among PPARs, each isoform possesses distinct biological activities [20] and is expressed in different tissues [19, 21, 22]. PPAR*α* is mainly expressed in the liver, the kidney, and the heart, and is primarily involved in lipid metabolism [22–24]. PPAR*γ* is a master regulator of adipogenesis and fat storage, which regulates adipocyte differentiation and insulin sensitivity in adipose tissue [22–24]. PPAR*β*/*δ* is found in a broad range of tissues with relatively high expression in brain, adipose tissue, and skin, but its function awaits further exploration [22–24]. Taken together, PPARs are key mediators of energy homeostasis, and lipid and glucose metabolism although they have also been associated with other biological processes including development, differentiation, inflammation, atherosclerosis, wound healing, and tumor formation [19, 21]. To date, PPAR*α* and PPAR*γ* are reported to possess significant clinical value. PPAR isoform-specific agonists, specifically fibrates for PPAR*α* and thiazolidinedione for PPAR*γ*, are currently prescribed as lipid and glucose-lowering drugs, respectively [23]. Interestingly, recent data have shown that expression of all three forms of PPARs displays circadian rhythm [22]. Hence, the interdependence of circadian and metabolic systems jointly regulated by PPARs appears to be disease relevant and thus, is the focus of this paper.

## 2. Reciprocal Regulation: Interplay between Central Circadian System and PPARs in Peripheral Tissues

PPARs act as molecular links between clock genes and specific rhythmic metabolic outputs. Emerging evidence has shown that, in peripheral tissues, PPARs and the core clock genes cross-regulate each other at transcription level. On one hand, the circadian expression of PPAR*α* is regulated by core clock genes and clock-controlled genes (Figure 1, (10)) [25–28]. On the other hand, it was shown that PPARs could directly affect circadian transcription of clock genes (Figure 1, (9)) [26, 29, 30]. The expanding regulatory network between PPARs and CLOCK proteins opens new perspectives for understanding the interdependence of PPARs and the core clock in peripheral tissues. 

### 2.1. The Master Clock Gene Products Regulate Transcription of PPARs

PPARs' circadian expression is controlled by the core clock gene products in peripheral tissues. CLOCK, one of the key components in the circadian regulatory network, directly controls the circadian expression of PPAR*α*. In wild-type mice, PPAR*α* expression displays diurnal variation in the liver [25, 31–34]. However, this rhythmic expression of PPAR*α* is abolished in the liver of CLOCK-mutant mice [25]. Furthermore, in contrast to the fibroblasts obtained from normal mice, fibroblasts from CLOCK-mutant mice exhibited decreased expression level and oscillation amplitude of PPAR*α* in response to oscillation inducer Endothelin-1 (ET-1) [35]. These findings indicate that CLOCK controls the circadian expression of PPAR*α* at the peripheral oscillator level. Sequence analysis revealed an E-box-rich region in exon 2 of the mouse PPAR*α* gene [25]. Chromatin immunoprecipitation (ChIP) of CLOCK in NIH3T3 cells indicates that CLOCK interacts with this E-box-rich region [25], suggesting that CLOCK might activate PPAR*α* transcription by interacting with E-Box in the exon 2 of PPAR*α*. Using a reporter construct constituting of E-box-rich region of PPAR*α* fused to luciferase, CLOCK and Bmal1 were shown to increase transcription by more than 25 folds [25]. E-Boxes are also present in human PPAR*α* gene (Figure 1, (10)) [25]. Moreover, Bmal1, another component of biological clock, was also shown to affect PPAR*α* circadian variation [26]. Furthermore, PPAR*α* mRNA expression was severely downregulated in the liver of *Bmal1* ^−/−^ mice. Collectively, these results suggest that PPAR*α* is a direct target gene of core clock proteins CLOCK/Bmal1. 

In addition to directly controlling the level of PPARs, CLOCK/Bmal1 can potentiate PPARs-mediated transcription activation. CLOCK/Bmal1 heterodimers have been shown to increase the transcriptional activity of genes whose promoter contains PPAR response elements (PPREs) [27]. Increased transcriptional activity was further enhanced by treatment with PPAR agonists (i.e., fenofibrate for PPAR*α* and troglitazone for PPAR*γ*) [27]. When PPRE was removed from the promoter, CLOCK/Bmal1 exhibited no effects on transcription [27], suggesting that the potentiation of transcription by CLOCK/Bmal1 is dependent on PPAR. However, the mechanism of CLOCK/Bmal1 and PPARs' synergistic regulation of gene expression remains to be explored. 

A recent paper provides a novel link between PPAR*β*/*δ* and Rev-erb*α*, an orphan nuclear receptor and central regulator of the clock gene. Rev-erb*α* was shown to maintain the circadian expression of mir-122, a microRNA abundantly found in hepatocytes, possibly via two ROREs in its promoter (Figure 1) [28]. mir-122 subsequently downregulates target genes by binding to a complementary sequence located in 3′ untranslated region (3′ UTR) [28]. Using reporter assays, mir-122 was shown to down-regulate PPAR*β*/*δ* [28]. Further investigation is needed to firmly establish the miRNA mediated regulation of PPAR*β*/*δ* by Rev-erb*α*.

### 2.2. PPARs Regulate the Peripheral Clock: A Role of Food Entrainment

PPARs are not only metabolic sensors but also circadian clock regulators. In the SCN of the PPAR*α*-null mice, clock genes (Bmal1, Per2, Per3, Cry2, and Rev-erb*α*) exhibited normal diurnal variation [26]. Moreover, bezafibrate, a PPAR*α* agonist, did not affect circadian expression of Per2, a core clock gene, in the SCN [29]. These findings indicate that PPAR*α* is not essential for maintaining the normal central clock oscillation. The peripheral clock, on the other hand, was affected by PPAR*α* expression. Changes in Bmal1 and Per3 expression was observed in the livers of PPAR*α*-null mice. Although the overall oscillation phase remained unperturbed, the amplitude of Bmal1 was decreased while Per3 amplitude was increased significantly [26]. Circadian rhythm in peripheral tissues such as liver can be reprogrammed, also termed entrainment, by feeding cycle alteration [36, 37]. Like other nocturnal animals, mice normally feed at night. Phases of the mouse liver PPAR*α* circadian expression could be inversed by daytime feeding [26]. In wild-type mice, clock genes (Bmal1, Per1, Per3, and Rev-erb*α*) showed inversed phase in response to daytime feeding corresponding to altered expression of PPAR*α* [26]. However, in PPAR*α*-null mice, although other clock genes can still be reset, Bmal1 was irresponsive to daytime feeding triggered by circadian reprogramming, suggesting that Bmal1 expression is controlled by PPAR*α* [26]. Furthermore, PPAR*α*-agonist fenofibrate could reset rhythmic expression and increase transcription of Bmal1, Per1, Per3, and Rev-erb*α* in livers of wild-type mice but failed to induce Bmal1 and Rev-erb*α* in livers of PPAR*α*-null mice, indicating that upregulation of Bmal1 and Rev-erb*α* by fenofibrate was mediated through PPAR*α* [26]. Another PPAR*α* agonist, bezafibrate, was shown to stimulate phase advancement of Per2 circadian expression in peripheral tissues [29]. ChIP analysis reveals that PPAR*α* directly binds to PPREs located at 1519 and 45 base pairs upstream of the transcription initiation site in Bmal1 and Rev-erb*α* promoters, respectively, [26]. Therefore, PPAR*α* could directly regulate transcription of Bmal1 and Rev-erb*α* via binding to PPREs in their respective promoter regions (Figure 1, (7)). Moreover, Per2 was shown to further enhance PPAR*α*-mediated activation of reporter gene fused to PPRE in a dose-dependent manner, suggesting that Per2 might function as a coactivator of PPAR*α* [17]. However, further investigations are necessary to validate this novel relationship.

Similar to its family members, PPAR*γ* can also modulate expression of biological clock components. PPAR*γ* rhythmic expression has been shown to precede that of Bmal1 in mice blood vessels [30]. Knocking down of PPAR*γ* abolishes rhythmicity of Bmal1, Cry1, Cry2, and Per2 in mice aorta [30]. The PPAR*γ*-agonist rosiglitazone induces Bmal1 expression (Figure 1, (9)) [30]. ChIP analysis reveals that PPAR*γ* also interacts with PPRE in Bmal1 promoter [30]. In reporter assays employing RORE-containing Bmal1 promoter segment, rosiglitazone treatment resulted in an increase in promoter activity, which was abolished when PPRE is mutated [30]. Taken together, PPAR*γ* appears to be a major regulator of Bmal1 expression in blood vessels.

In summary, the central circadian clock components and PPARs exhibit a reciprocal regulation. Circadian clock proteins control PPARs expression by at least two mechanisms: (1) activation of PPAR*α* transcription by CLOCK/Bmal1 heterodimer upon binding to the E-box in the PPAR*α* promoter and (2) downregulation of PPAR*β*/*δ* by microRNA stimulated by Rev-erb*α*. On the other hand, PPARs regulate the expression of clock genes via two different mechanisms as well: (1) Rev-erb*α* is positively regulated by PPAR*α*; (2) Bmal1 is positively regulated by both PPAR*α* and PPAR*γ*. The multivariable regulatory loops of the core clock proteins and PPARs are diagrammatically summarized in Figure 1.

### 2.3. PPAR Cofactors in Circadian Rhythm

#### 2.3.1. NcoR1 and Hdac3

In the absence of ligand, PPARs form a multicomponent complex with corepressors, such as nuclear receptor corepressor 1 (NcoR1) [38]. Histone deacetylase 3 (Hdac3), a histone modification enzyme, is recruited and stably bound to the repressor complex through a conserved deacetylase activation domain (DAD) in NcoR1 [39]. Hdac3 remodels chromatin in a way less favorable for access by basal transcriptional machinery, leading to the repression of PPAR-mediated transcription activation [38]. NcoR1 is directly involved in the biological clock system as a cofactor for Rev-erb*α*. Upon binding to RORE in the Bmal1 promoter, Rev-erb*α* recruits NcoR1/Hdac3 to suppress expression of Bmal1 [40]. NcoR1 and Hdac3 are critical for the regulation of clock genes and energy metabolism homeostasis. A mouse line was generated to harbor a mutation in NcoR1 DAD domain that abolished the ability of NcoR1 to interact with or activate Hdac3 [39]. In NcoR1 mutant knock-in mice, uncoupling of NcoR1 from binding to Hdac3 led to altered circadian rhythmicity [39]. Bmal1 expression level was higher in the mutant mice than that of wild-type [39]. This is consistent with the established corepressor function of NcoR1/Hdac3 for Rev-erb*α* which downregulates Bmal1. Circadian expression abnormality of Bmal1 and Rev-erb*α* was also observed in these mutant mice [39]. Moreover, the mutant mice exhibited altered expression patterns of liver genes involved in lipid metabolism. Phase shifts were observed with genes harboring fat catabolism function such as carnitine palmitotransferase 1a (Cpt1a), median chain aryl-CoA dehydrogenase (MCAD), and their regulator PPAR*α* [39]. ATP citrate lyase (Acly) and acetyl CoA carboxylase2 (Acc2) displayed phased reversion [39]. Expression level of elongation of long-chain fatty acids family member 6 (Elovl6) was decreased by fourfold in mutant mice as compared with wild-type mice [39], which might explain the increased leanness and insulin sensitivity. The mutant mice demonstrated distinct, seemingly desirable phenotype including increased leanness, decreased body weight, reduced body fat, increased O_2_ consumption, increased body heat generation, and improved insulin sensitivity [39]. Thus, uncoupling NcoR1/Hdac3 interaction could be beneficial for metabolic syndrome management.

#### 2.3.2. PPAR*γ* Coactivator 1*α* (PGC-1*α*)

It is believed that ligand binding to PPAR triggers a conformational change which allows the dissociation of corepressors and recruitment of coactivators that many of which are known to be histone acetyltransferases (HATs) [41], resulting in transcription activation of target genes. PPAR*γ* coactivator-1*α* (PGC-1*α*), as the name implies, is a known coactivator of PPARs [20]. In addition to PPARs, PGC-1*α* is capable of activating other nuclear receptors such as thyroid receptor b, estrogen receptor and glucocorticoid receptor TR*β*, ER, and GR [42].

PGC-1*α* is well characterized to be a part of the biological clock. PCG-1*α* positively regulates the expression of clock components, including CLOCK, Bmal1, and Rev-erb*α* [43]. PGC-1*α* can physically interact with ROR*α* and ROR*γ* through the LXXLL motif and enhance ROR*α* transcription activity [43]. When PGC-1*α* is recruited to the ROR-bound promoter, it could recruit p300 and GCN5 histone acetyltransferase to modify local chromatin structure to be permissive to transcriptional machinery [43]. On the Bmal1 promoter, PGC-1*α* binding is accompanied by an increase in histone H3 acetylation and histone 3 lysine 4 trimethylation (H3K4me3), two markers of transcriptional activation, while histone 3 lysine 9 dimethylation (H3K9me2), a marker that signifies transcription silencing, decreased [43]. Taken together, PGC-1*α* is a coactivator of ROR*α* which converts chromatin from a quiescent to transcriptionally permeable state thus enhancing transcription. Disruption of PGC-1*α* leads to alteration in locomotor behavior, O_2_ consumption, and expression pattern of metabolic genes and clock genes circuitry [43]. Thus, PGC-1*α* serves as a potential factor which couples circadian rhythm to energy status.

## 3. Medical Implications: Metabolic Syndrome and Other Diseases Potentially Linked to the Aberrant PPAR Circadian Rhythm

### 3.1. Circadian Oscillation of PPARs Displays a Strong Association with the Energy and Metabolism Homeostasis

PPARs play essential functions in energy homeostasis. Circadian oscillation of PPARs is essential for the temporal coordination of genes involved in energy and metabolic processes. PPAR*α* exerts a strong impact on lipid metabolism. Disruption of PPAR*α* resulted in alteration of the circadian expression of these metabolism-related genes [44]. Interestingly, the expression patterns of the lipolytic genes appear to oscillate in-phase with those of PPAR*α*, while those of the lipogenic genes oppose the expression pattern of PPAR*α*. In mouse, the mRNA level of cytosolic acyl-CoA thioesterase (CTE-I), an enzyme catalyzing lipid hydrolysis, exhibits diurnal rhythm parallel to the circadian PPAR*α* expression. The expression of CTE-I can be induced by fasting; that is the expression is increased during the light phase and declined during the dark phase when feeding activity is abundant [44]. The fasting-induced CTE-I mRNA level is lower in PPAR*α*-null mice than that in the normal mice [44], suggesting that CTE-I diurnal rhythm is regulated through PPAR*α*. In wild-type but not in PPAR*α*-mutant mice, the administration of the PPAR*α*-agonist bezafibrate could induce circadian expression of fibroblast growth factor 21 (FGF21) [34], a hormone involved in lipolysis and hepatic ketogenesis [45], suggesting that bezafibrate-induced circadian effect is strictly PPAR*α*-dependent.

On the contrary, the expression of enzymes involved in lipid synthesis such as fatty acid synthase (FAS) and acetyl Co-A decarboxylase (ACC) in the fatty acid synthesis pathway and 3-hydroxyl-3-methylglutaryl-CoA reductase (HMG-CoAR) in cholesterol synthesis pathway oppose the expression patterns of PPAR*α*. These enzymes exhibited increased expression during dark phase in mice [32]. Again, the circadian expression of these enzymes is abrogated in PPAR*α*-null mice [32], indicating that the diurnal variation of these enzymes requires PPAR*α*.

There is evidence suggesting that the feeding behavior is mediated, in part, by PPAR*α*. Oleoylethanolamide (OEA), a naturally-produced lipid compound found in mammals including humans, is a satiety stimulator [46]. Later, OEA was shown to be a PPAR*α* agonist and displayed circadian expression [47]. Administration of OEA analogues can suppress feeding and decrease weight gain in wild-type mice [47]. However, this response was not observed in PPAR*α*-null mice [47], indicating that OEA regulates satiety through activation of PPAR*α*. Nonetheless, the detailed mechanism by which OEA regulates circadian oscillation remains to be elucidated.

PPAR*β*/*δ* has been linked to regulation of body temperature and lipid profile. PPAR*β*/*δ* activates genes involved in fatty acid oxidation, resulting in increased lipolysis in adipocyte and skeletal muscle cells [48]. Expression of PPAR*β*/*δ* triggers lipolysis in brown adipose tissue, a body compartment which is central to adaptive thermogenesis, during which processed energy is dissipated as heat via uncoupling proteins (UCPs) [48]. PPAR*β*/*δ* expression cycle has been shown to oscillate in-phase with that of uncoupling protein 1(UCP1) [22], suggesting that PPAR*β*/*δ* could promote energy dissipation. However, this relationship needs to be validated experimentally.

In conclusion, PPARs serve as sensors which integrate energy and metabolic homeostasis to circadian clock. Therefore, the aberration of clock genes could result in altered expression of metabolic genes, leading to disturbance of energy status in affected organisms. This imbalance is known to attribute to metabolic syndrome, a complex disease with distinct hallmarks including obesity, dyslipidemia, hypertension, and elevated plasma glucose level [49]. Further experiments investigating this relationship could deepen our understanding of pathogenesis, which may pave ways for new strategies to fight against metabolic syndrome.

### 3.2. Effect of Gender on the Biological Clock and PPARs Expression

Clinically, patients with comparable health are usually administered the same treatment regimen, regardless of gender. However, with the emerging idea of individualized medication, gender might need to be taken into account. Experiments in mice revealed distinct expression patterns of clock genes in the liver and hepatic lipid homeostasis differences between males and females [50]. The majority of hepatic clock genes including CLOCK, Bmal1, Per1, Per2, Cry1, and Rev-erb*α* reached mRNA peaks 30 minutes earlier in female as compared to male mice, while Cry2 and Per3 peaked earlier in male than female mice [50]. Statistically calculated mean expression levels of Per1, Per3, and ROR*α* were also higher in female mice [50] as well as the amplitude of Cry1 and Per3 [50]. Similarly, PPARs and their coactivators showed differential expression between the two genders. PPAR*α*, PPAR*β*/*δ*, and PGC-1*β* expression peaked at least 30 minutes earlier in female mice [50]. Statistically calculated mean expression levels of PPAR*α*, PGC-1*α* and PGC-1*β* were higher in female mice than the male counterparts [50]. Moreover, the expression pattern of genes involved in lipid metabolism, triglyceride and cholesterol profiles in liver and serum of male and female mice fluctuated differently throughout the day [50]. Taken together, these findings implicate gender associated disparity of clock gene rhythmicity, expression patterns of genes involved in energy homeostasis, and serum hepatic lipid profile. Since these differences could potentially affect the efficacy of drugs that target circadian clock and lipid metabolism, several issues are worth noting. Should drug administration be tailored specifically for males and females based on these differences? Can this intervention increase efficacy or reduce untoward effects of mediation? Further studies would be necessary to provide concrete solutions.

### 3.3. Other Circadian Physiology with PPARs Involvement: Stress, Sleep-Awake Cycle, and Blood Pressure

Circadian clocks essentially regulate rhythmic cellular and physiological processes, providing a platform for communications among different physiological processes. It is worthy of noting that PPARs are among a number of nuclear receptors that mediate the connections between circadian clocks and physiological processes. The functions of nuclear receptors in regulating circadian clock and physiology have been extensively discussed in several recent papers [51, 52] thus this paper will focus on PPARs. Besides their major roles in regulating lipid, glucose, and energy metabolisms, PPARs are also involved in regulating behavior rhythm and other physiological rhythms such as body temperature, blood pressure, and sleep phase. 

As the endocrine system is also involved in clock entrainment by metabolic cues, there is functional crosstalk between PPARs and endocrine system. Aside from the nutrient status, PPAR*α* level is regulated by oscillation of steroid hormones. In mice, PPAR*α* gene expression is positively controlled by glucocorticoids [31, 53, 54], stress hormones whose secretion displays diurnal rhythm [31]. Mice PPAR*α* mRNA and protein levels were also shown to oscillate in-phase with plasma corticosterone [31], suggesting that PPAR*α* might play a role in the stress response. 

PPAR*α* has been implicated in regulating behavior rhythm such as sleep-wake cycle. Locomotor activity of mice with delayed sleep phase syndrome (DSPS), a disease described by persistent delayed sleep onset, can be restored by bezafibrate administration, implicating that bezafibrate may be a putative drug for DSPS treatment [29].

PPARs are also sensors of environmental cues to orchestrate distinct physiological rhythms such as body temperature, heart rate, and blood pressure. Mice with PPAR*γ* deletion exhibits abnormal blood pressure and heart rate circadian rhythm, in accordance with decreased diurnal variation in sympathetic activity [30]. Alteration in circadian expression pattern of vascular Bmal1 has been observed in these mice [30], implicating the involvement of Bmal1 in cardiovascular physiology.

In summary, the biological clock is a self-sustained regulatory system which, under normal circumstance, allows appropriate acclimation of body physiology such as metabolic rate, blood pressure, and alertness to the immediate environment. In order to maintain homeostasis, environmental factors, by large light and food, trigger signal transduction to synchronize the circadian clock resulting in appropriate expression of downstream genes. Our paper exemplifies the bidirectional regulatory loop between PPARs and circadian clock. Together, PPARs and the biological clock play roles in maintenance of the expression of metabolic genes in response to the surrounding environment. Due to its strong association to energy status, deregulation of the PPARs-circadian clock system is believed to contribute, at least in part, to the development of metabolic syndrome. Furthermore, emerging evidence also suggests that disturbance in the PPAR-circadian clock system could affect various aspects of physiology including stress response, blood pressure, and sleep-awake cycle (Figure 2). Thus the disease link between PPARs and the circadian clock has become an exploratory area of PPARs research.

## 4. Conclusion

Circadian rhythm is essential for coordination of physiology and behavior in living organisms to respond to the immediate environment in a timely manner. Specialized proteins including CLOCK, Bmal1, Per, Cry, ROR*α*, and Rev-erb*α* are responsible for the assembly of a complex regulatory system that possesses self-sustained circadian oscillation capacity in the circuit. PPARs in peripheral tissues exhibit a strong interplay with the central circadian clock components, serving both as affecters and effecters of the clock system. We diagrammatically summarize the biological clock transcriptional regulatory networks (Figure 1) and the functional roles of PPARs (Figure 2) in the context of circadian regulation. Since the major function of PPARs is to regulate metabolism and energy homeostasis, it seems plausible that PPARs are critical players to coordinate energy status of an organism to the central biological clock. Thus, dissecting the functional roles of PPARs in circadian rhythm could potentially advance our understanding on mechanisms of disorders in energy homeostasis and metabolism.
